# Recovery-focused mental health care planning and co-ordination in acute inpatient mental health settings: a cross national comparative mixed methods study

**DOI:** 10.1186/s12888-019-2094-7

**Published:** 2019-04-16

**Authors:** Michael Coffey, Ben Hannigan, Sally Barlow, Martin Cartwright, Rachel Cohen, Alison Faulkner, Aled Jones, Alan Simpson

**Affiliations:** 1 Department of Public Health, Policy and Social Sciences, Swansea University, Singleton Park, Swansea, SA2 8PP UK; 2 School of Healthcare Sciences, Cardiff University, Cardiff, CF24 0AB UK; 3 Centre for Mental Health Research, School of Health Sciences, City, University of London, Northampton, Square, EC1V 0HB UK; 4 Centre for Health Services Research, School of Health Sciences, City, University of London, Square, EC1V 0HB, Northampton, UK; 5 Centre for Academic Mental Health, Bristol Medical School, University of Bristol, Oakfield House, Oakfield Grove, Clifton, Bristol, BS8 2BN UK; 6 Independent Service User Researcher Consultant, London, UK; 7 East London NHS Foundation Trust, 9 Alie St, London, E1 8DE UK

**Keywords:** Acute, Care planning, Inpatient care, Personalisation, Recovery, Risk, Therapeutic relationships

## Abstract

**Background:**

Involving mental health service users in planning and reviewing their care can help personalised care focused on recovery, with the aim of developing goals specific to the individual and designed to maximise achievements and social integration. We aimed to ascertain the views of service users, carers and staff in acute inpatient wards on factors that facilitated or acted as barriers to collaborative, recovery-focused care.

**Methods:**

A cross-national comparative mixed-methods study involving 19 mental health wards in six service provider sites in England and Wales. This included a survey using established standardised measures of service users (*n* = 301) and staff (*n* = 290) and embedded case studies involving interviews with staff, service users and carers (*n* = 76). Quantitative and qualitative data were analysed within and across sites using descriptive and inferential statistics, and framework method.

**Results:**

For service users, when recovery-oriented focus was high, the quality of care was rated highly, as was the quality of therapeutic relationships. For staff, there was a moderate correlation between recovery orientation and quality of therapeutic relationships, with considerable variability. Staff members rated the quality of therapeutic relationships higher than service users did. Staff accounts of routine collaboration contrasted with a more mixed picture in service user accounts. Definitions and understandings of recovery varied, as did views of hospital care in promoting recovery. Managing risk was a central issue for staff, and service users were aware of measures taken to keep them safe, although their involvement in discussions was less apparent.

**Conclusions:**

There is positive practice within acute inpatient wards, with evidence of commitment to safe, respectful, compassionate care. Recovery ideas were evident but there remained ambivalence on their relevance to inpatient care. Service users were aware of efforts taken to keep them safe, but despite measures described by staff, they did not feel routinely involved in care planning or risk management decisions. Research on increasing therapeutic contact time, shared decision making in risk assessment and using recovery focused tools could further promote personalised and recovery-focused care planning.

This paper arises from a larger study published by National Institute for Health Research (Simpson A, et al, Health Serv Deliv Res 5(26), 2017).

**Electronic supplementary material:**

The online version of this article (10.1186/s12888-019-2094-7) contains supplementary material, which is available to authorized users.

## Background

Improving the treatment and care of people with mental illness is amongst key priorities for health and social care in both England and Wales [1]. However, despite the shift to community-based models of care, considerable resources are still spent on acute inpatient beds: as much as £585million in 2009–10 [2].

In England in 2016–17, 101,589 people in contact with mental health and learning disability services spent time in hospital, with an estimated 45,864 people detained under the Mental Health Act (MHA) 1983 [3]. In Wales, 8723 admissions to hospital for mental illness took place in 2016–17, with 1776 of these taking place using sections of the MHA 1983 [4]. This volume of admissions requires considerable planning and coordination to ensure effective care is delivered consistently.

Health care is a devolved responsibility in the UK meaning that the context and delivery of mental health care is diverging between countries, providing a rich geographical comparison for research. In England the care programme approach (CPA), and in Wales care and treatment plans (CTPs), oblige providers to: comprehensively assess health/social care needs and risks; develop a written care plan; allocate a care co-ordinator; and regularly review care. CPA/CTP processes are now also expected to reflect a philosophy of recovery and to promote personalised care [5, 6]. These similarities between CPA and CTP mask an important difference too however. CPA in England is central guidance while CTP in Wales is legislative and places legal obligations on health boards and local authorities. CTP in Wales uniquely has an associated code of practice, stipulating for example that only specifically qualified workers (e.g. registered mental health nurses, occupational therapists and clinical psychologists) can act as care co-ordinators [7].

The concept of recovery in mental health was initially developed by service users and refers to “a way of living a satisfying, hopeful, and contributing life even with limitations caused by illness,” while developing new purpose or meaning [8].^(p527)^ The importance of addressing personal recovery, alongside more conventional ideas of clinical recovery [9] is now supported in guidance for all key professions [10–13]. To this has been added the idea of personalisation. This aims to see people and their families taking more control over their support and treatment options, alongside new levels of partnership and collaboration between service users (or citizens) and professionals [14].^(p3)^ Recovery and personalisation in combination mean tailoring support to fit the specific needs of the individual and enabling social integration through greater involvement of local communities [15].

The CPA/CTP are central to modern mental health care [16] yet there are few studies that explicitly explore the practices of care planning and coordination in community services and even fewer focusing on inpatient care planning [17]. A relatively rare example of the former is the recently completed COCAPP study [18, 19]. In the UK national quality statements include the requirement that service users can jointly develop a care plan with mental health professionals, are given a copy with an agreed date to review it, and are routinely involved in shared decision-making [20]. National policies [1, 6] outline expectations of recovery and involvement in decisions about treatment. This holds true for both informal and detained inpatients, with a requirement that reasonable adjustments are made where necessary to ensure that people are supported to live as full and socially participative lives as possible [21]. However, national quality reviews reveal limited evidence of service users’ views being listened to, with concerns being raised that control and containment are prioritised over treatment and support [21].

Earlier national reviews across both nations found that service users remained largely mystified by the care planning and review process itself, with significant proportions not understanding their care plans, not receiving written copies of their plan and often not feeling involved in the writing of care plans and setting of goals [22, 23]. Clearly, there are significant problems with inpatient care planning with the Care Quality Commission (CQC) noting “significant gap between the realities observed in practice and the ambitions of the national mental health policy” [21].^(p5)^ The House of Commons Health Committee [24] subsequently reported widespread concerns about delays in care planning and an imbalance between a focus on risk rather than recovery.

Previously, the Healthcare Commission [25] measured performance on 554 wards across 69 NHS Trusts providing mental health acute inpatient services. They found that almost two-fifths of trusts (39%) scored weak on involving service users and carers; 50% of care plans sampled did not record the service user’s views; and nearly a third of care records (30%) did not record whether or not the service user had a carer. A third of all care records sampled (33%) showed that community care coordinators provided input into the service users’ care review meetings only “some or none of the time”.

## Aim

The aim of this study was to identify factors that facilitate or hinder recovery-focused personalised care planning and coordination in acute inpatient mental health settings. As an exploratory study guided by the Medical Research Council (MRC) [26] Complex Interventions Framework we aimed to generate empirical data, new theoretical knowledge and greater understanding of the complex relationships between collaborative care planning, recovery and personalisation.

## Methods

### Design

We conducted a cross-national comparative study of recovery-focused care planning and coordination in inpatient mental healthcare settings, employing a concurrent transformative mixed methods approach with embedded case studies [27]. A full account of our methods is provided elsewhere [28].

In summary, our study was informed by systems ideas emphasising connections between macro, meso and micro levels of organisation [29]. Cross-national comparative research involves “comparisons of political and economic systems …and social structures” [30] ^(p93)^ where “one or more units in two or more societies, cultures or countries are compared in respect of the same concepts and concerning the systematic analysis of phenomena, usually with the intention of explaining them and generalising from them” [31]. ^(p1–2)^ In this study, devolved government and the emergence of similar but distinct health policy, legislation and service development in England and Wales provided the macro-level national context.

**Fig. 1:**
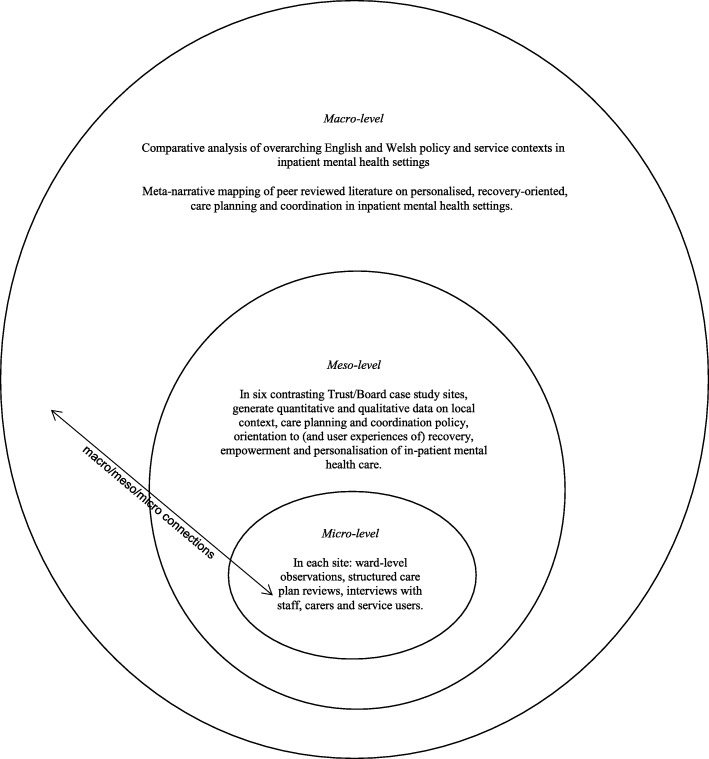
Diagram illustrating embedded case study design and integration of care planning and coordination in acute inpatient mental health settings

A case study method [32] allows the exploration of a particular phenomenon within dynamic contexts where multiple influencing variables are difficult to isolate [33]. It allows consideration of historical and social contexts [34] and is especially useful in explaining real-life links that are potentially too complex for survey or experimental approaches [35]. The definitions of the case studies were predetermined [36], focusing on selected NHS Trust/Health Boards. Data collection at this meso-level included identifying local policy and service developments alongside empirical investigations of care planning and inpatient care, recovery, personalisation, therapeutic relationships and empowerment, employing mixed quantitative and qualitative methods. This design is represented in Fig. 1.

### Sampling

We selected six case study sites to match our earlier community study [18] so that comparisons and connections between community and inpatient services could be drawn [32]. These consisted of four NHS Trusts in England and two Local Health Boards in Wales that are commissioned to provide inpatient mental health services. In total 19 acute wards were selected for data collection. These sites reflected a mix of rural, urban and inner city settings in which routine inpatient care is provided to people with complex and enduring mental health problems. In each site, a single acute inpatient ward was chosen for further in-depth investigation and up to six service users, six multidisciplinary staff and four informal carers were sampled as embedded micro-level case studies [27]. Inclusion criteria for wards included that these were providing acute mental health care admissions facilities to the local adult population and had an established ward manager/team leader in post. Inclusion criteria for service user participants included that they were currently admitted to the in-patient facility, had been on the ward for a minimum of 7 days, 18 years or older, with a history of severe mental illness and able to provide informed consent. Staff inclusion criteria were staff working on inpatient wards involved in care planning or review. Full inclusion and exclusion criteria are provided elsewhere [28].

### Sample size calculations

For the survey, an a priori sample size calculation was conducted using the G*Power software (version 3.1) [37]. The estimated sample size for service users was calculated for the global effect of a one-way multivariate analysis of variance (MANOVA) with six groups (sites), 17 outcomes (Recovery Self-Assessment Scale total (+ 5 sub-scales), Scale To Assess the Therapeutic Relationship total (+ 3 sub-scales), Empowerment Scale total (+ 5 sub-scales) and the Views of Inpatient Care Scale total), an α level of 0.05, power of 0.8 and a small effect size (f^2^ = 0.029). This calculation suggested that a total of 276 service user participants was required.

We anticipated that with non-response and incomplete measures we would need to oversample, we therefore decided to recruit 300 service users (*n* = 50 per Trust/Health Board) and 300 inpatient staff (*n* = 50 per site). We anticipated that we would not achieve this sample size for informal carers and therefore aimed to recruit 150 informal carers (*n* = 25 per Trust/Health Board). This was because not every service user would have a carer, therefore analysis for the informal carers would be underpowered (estimated power was 0.44). The data for the informal carers was therefore anticipated to be exploratory.

Sample size calculations for qualitative interviews were based on previous research with similar populations by the co-investigators and others. Calculations were based on understanding of the practicalities and time commitments of recruiting and interviewing participants and analysing in-depth qualitative data; and the numbers required to feel confident that the findings would be transferable to other similar settings.

### Instrumentation

The data collection measures reported in this paper are;

1. The Recovery Self-Assessment Scale (RSA) [38]: a 36-item scale measuring the extent of recovery-oriented practices. The scale addresses the domains of life goals, involvement, treatment options, choice and individually tailored services. Acceptable internal consistency of the RSA with Cronbach’s alpha has previously been demonstrated [18]. It was completed by service users, carers and ward staff. In the current study Cronbach’s alpha for the Total RSA scale for service users was 0.98 (*N* = 103) and for staff was 0.95 (*N* = 186); Life Goals subscale, for service users was 0.93 (*N* = 179) and for staff was 0.86 (*N* = 246); Involvement subscale, for service users 0.91 (*N* = 163) and for staff was 0.85 (*N* = 225); Diversity of Treatment options subscale, for service users was 0.81 (*N* = 172) and for staff was 0.77 (N = 225); Choice subscale, for service users was 0.81 (*N* = 217) and for staff was 0.68 (*N* = 254) and Individually Tailored Services subscale for service users was 0.85 (*N* = 159) and for staff was 0.71 (*N* = 253).
2. The patient and clinician versions of the Scale To Assess the Therapeutic Relationship (STAR-P and STAR-C) [39]: a 12-item scale assessing therapeutic relationships. A total STAR score is obtained by summing individual items. The subscales measure positive collaborations (possible scores 0–24), positive clinician input (possible score 0–12) and non-supportive clinician input in the patient version and emotional difficulties in the staff version (possible score 0–12). It was completed by service users and ward staff. Cronbach’s alpha for the total STAR-P scale for service users was 0.89 (*N* = 264) and for staff was 0.81 (*N* = 263); Positive Collaboration subscale, for service users was 0.92 (*N* = 279) and for staff was 0.81 (*N* = 269); Positive clinician input subscale, for service users was 0.72 (*N* = 282) and for staff was 0.56 (*N* = 268); and Non-Supportive clinician input subscale, for service users was 0.67 (*N* = 284) and for staff was 0.63 (*N* = 273).
3. The Empowerment Scale (ES) [40]: a 28-item questionnaire with five subscales: self-esteem, power, community activism, optimism and righteous anger. A total empowerment score is obtained by summing individual items and dividing them by the number of items. Subscale values can also be provided for ‘self-esteem-self-efficacy’, ‘power-powerlessness’, community activism and autonomy’, ‘optimism and control over the future’ and ‘righteous anger’. This scale was completed by service users. Cronbach’s alpha for the total Empowerment scale for service users was 0.82 (*N* = 255); Self-esteem-self-efficacy subscale, 0.91 (*N* = 272); Power-Powerlessness subscale 0.56 (*N* = 271); Community activism and autonomy subscale, 0.58 (*N* = 276); Optimism and control, 0.70 (*N* = 275) and Righteous anger, 0.40 (*N* = 281).
4. The Views of Inpatient Care Scale (VOICE) [41]: a 19-item patient-reported outcome measure of perceptions of acute mental health care that includes questions on involvement in care planning and ward round discussions. VOICE total score was obtained by summing individual item scores, possible total scores range from 19 to 114. The higher the total score for the VOICE the more negative the perception of the quality of care on the ward. It was completed by service users.

We further investigated internal consistency using alternative approaches, mean item-total correlations and Spearman-Brown prediction values (see Additional file 1). These additional analyses suggested that all subscales had acceptable internal consistency, although two subscales of the Empowerment Scale would merit further psychometric development.

We additionally conducted semi-structured interviews with ward staff, service users and carers. Interview schedules were based on our previous study and refined in consultation with our Scientific Steering Committee and Lived Experience Advisory Group (LEAG) and drawing on relevant literature. The aim of all interviews was to explore participants’ views and experiences of care planning and co-ordination, safety and risk, recovery and personalisation, and the context within which these operated. Care plan reviews and observations of ward rounds were also conducted but are not reported in this paper. In some cases participants on the case study sites completed surveys and research interviews but this was not a requirement of the study and the majority chose to participate in one part of the study only.

### Research ethics

The study received NHS Research Ethics approval from the NRES Committee NRES Committee London – Fulham (Ref: 13/LO/2062) on 29th December 2014.

Considerable attention was given to ensuring the welfare of service user, carer and other participants and of the researchers. This included providing opportunities to pause or withdraw from interviews, assurances of anonymity and confidentiality and responding to concerns for people’s welfare.

### Public and patient involvement (PPI) and study oversight

The study was developed and designed with full involvement of co-investigator and independent service user researcher (AF) and in consultation with SUGAR (Service User and Carer Group Advising on Research [42]). In addition, a Lived Experience Advisory Group (LEAG) met every 4/6 months during the study, consisting of seven service users and one carer with direct experience of inpatient mental health care.

The 12-member independently chaired Scientific Steering Committee (SSC) consisted of representatives with a clinical or research background from each of the participating NHS Trusts/Health Boards, as well as independent academics. One service user and one carer member also represented the LEAG.

Three Service User Researcher Assistants (SURAs)/Service User Project Assistants (SUPAs) were employed to recruit participants and conduct research interviews. All received training and ongoing support throughout the study.

### Procedure

Suitable local wards meeting inclusion criteria were identified with the assistance of local NHS Trust/Health Board principal investigators. Ward managers were approached by a researcher who explained the study, responded to any queries and invited them to participate. No service declined to take part. We sought approval to participate from two or three wards in each area and one of the three wards was then selected for in-depth case study of care planning including interviews. Each site was given a pseudonym to help maintain anonymity of participants. French names were chosen to avoid any accidental connection with English or Welsh sites or regions. The site names are:

### Survey

All managers and ward staff involved in care planning or care plan review received written and verbal information about the study and were invited to participate in the survey (target n = 50 per Trust/Health Board).

Staff from participating wards were asked to identify service users who had been on that ward for a minimum of seven days, and who in their view potentially had the capacity to participate in the study. The service user was provided with written and verbal information by a researcher, who then ensured the person was able to provide informed consent to participate. Each participant was then given a survey pack to complete, with assistance if required. A thank you gift of £10 was given to service user participants on completion of the survey pack.

Ward staff were asked to give carer survey packs to carers (family members and friends) visiting service users on the ward (target *n* = 25 per Trust/Health Board). The packs included an information sheet and a Freepost return envelope. Researchers working on the ward also approached carers to invite them to participate by completing measures.

### Semi-structured interviews

Key personnel (registered nurses, ward managers, occupational therapists, psychologists and psychiatrists) were identified using purposive sampling to reflect meso and micro level functions. They were invited to participate in research interviews for the in-depth case study (target *n* = 6 per case study ward; total *n* = 36). Micro-level refers to the level at which face-to-face care is organised, provided and received. For our purposes meso-level refers to management functions that enable or structure micro-level work. Staff were given written materials describing the purpose of the study including the option to decline or withdraw at any time. Informed consent procedures were followed.

Service users approaching discharge were invited to participate in an interview about their experiences of care planning and jointly review their care plan (target *n* = 6 per case study ward; total *n* = 36). Informed consent procedures were followed. A thank you gift of £10 was given to service user participants on completion of the research interview.

Service users were asked to identify a carer (if applicable) to take part in an interview (target *n* = 4 per case study ward; total *n* = 24). Carers were contacted by telephone or when visiting, in the presence of the service user if possible. Informed consent procedures were followed.

### Data management and analysis

Qualitative and quantitative data in each of the sites were considered on a within-group basis prior to a cross-case analysis aimed at identifying common themes and divergences. The between-group analysis of the quantitative data compared service users and staff across sites on key markers of the service user experience (recovery-oriented care, therapeutic relationship and empowerment). The quantitative analyses were conducted alongside the qualitative analyses in a convergent parallel design that facilitates the integration of mixed methods data [27]. Large scale survey data provides a broad picture while the interview data offers more micro detail. This is a pragmatic approach to mixed method research that can generate a more complete understanding of complex phenomena or processes. Quantitative and qualitative data analyses were conducted independently and subsequently synthesised to generate understanding of the links across micro, meso and macro levels than either approach could achieve alone.

### Quantitative data

Data from the questionnaires were entered into SPSS version 21 [43] and distribution of the data assessed for normality using descriptive quantitative measures of skewness and kurtosis. There were few deviations from normality (2 of 27 scale outcomes exceeded the conservative criteria of +/− 1), one was small in the extent of deviation (within +/− 2) however one scale displayed larger deviation of skewness (Emotional differences subscale, Staff outcome on the STAR-C).

A missing value analysis was completed for the 27 scale outcomes. Moderate to high levels of missing data, not missing at random, were identified on a small number of items (mean level of missing data across the 27 scales/subscales was 20%, range from 6 to 55%). The service user version of the RSA questionnaire in particular had a moderate amount of missing data. Mean replacement was used to avoid unnecessary loss of cases from the analysis. The mean of the available items for the scale and participant were used for replacement of the missing values on the scale. A series of sensitivity analyses were conducted to determine what effect mean replacement would have in the primary analyses at different levels of replacement ranging from 20 to 50% replacement. Utilising a 50% mean replacement had no substantive changes in the key statistical parameters (*p*-values and associated effect sizes) and the inferences drawn, therefore it was deemed appropriate to maximise the number of cases included in the analyses.

Descriptive statistics were calculated for the four measures (VOICE, RSA, STAR and ES). Where appropriate these scores were compared against reference values (VOICE, STAR and ES) or to the participant groups (RSA). Several unadjusted one-way Analysis of Variance (ANOVA) were conducted to compare differences between the six sites on the RSA, STAR, ES and VOICE measures. Subsequent Tukey post hoc tests were conducted to ascertain which measures differed between which locations. A series of one-way analysis of covariance (ANCOVA) were completed to adjust the analyses for potential confounders. The demographic variables that were chosen for service users were: age; gender; ethnicity and living status. Three care-related variables were chosen for service users: previous admissions; time in mental health services and time on the ward. The demographic variables that were chosen for staff were: age, gender, ethnicity, personal experience of mental illness and family experience of mental illness. Two clinical variables were also chosen: time working in mental health services and time working on the ward. The criteria for adjusted analysis between the ANOVA and ANCOVA were the *p*-value from the omnibus test, the adjusted means and the p-value from the post-hoc test. If the p-value from the omnibus test for the ANCOVAs were not substantively different from the ANOVAs then no further post –hoc analyses were completed. A series of independent t-tests were completed to determine if there were differences between service users and staff on the outcome measures.

Correlations of the service user data were completed to identify if there was a relationship between the scores on the outcome measures used. Six Pearson’s correlations were conducted to identify if there were relationships between the mean total scores for the measures RSA and VOICE; RSA and STAR-P; RSA and ES; STAR-P and ES; STAR-P and VOICE and VOICE and ES for all service user participants and by individual site. Cohen’s [44] effect sizes were used to describe the data (Small, *r* = 0.10, medium *r* = 0.30 and large *r* = 0.50). A Pearson correlation was also completed for staff on the mean total scores for the RSA and STAR-C.

For all the ANOVA and ANCOVA analysis the statistical significance level was set at a level of 0.05. To account for multiple comparisons for the t-tests the significance threshold was raised to 0.005 to accommodate for the number of tests applied (*n* = 10).

### Qualitative data

All digital interview recordings were professionally transcribed and checked against original recordings for accuracy and identifying information redacted, before being imported into QSR International’s NVivo10 qualitative data analysis software [45] for analysis using Framework method [46, 47]. The Framework matrix used was developed a priori from the interview schedules, with sections focusing on organisational background and developments, care planning, recovery, personalisation, safety and risk, and recommendations for improvement. Each matrix section also had an ‘other’ column for the inclusion of data-led emergent categories. Once all charting was completed, second-level summarising was undertaken to further *précis* data and to identify commonalities and differences.

## Results

Data collection across the six sites is summarised in Table 1 and consisted of *n* = 301 service users (target was 300), *n* = 290 members of staff (target was 300), *n* = 28 carers (target 150) completing survey measures.

**Table 1:** Summary site characteristics and data collection across the six meso-level case study sites

Site (country)	Characteristics of the Site	Questionnaire Returns		Interviews		
		Staff	Service Users	Staff	Service Users	Carers
Artois (England)	Covers a large and predominantly rural area, serving a population of around 1.6 million. There are 8 adult psychiatric admissions wards with 157 beds available. The main ward for intensive data collection was mixed gender and had 23 beds: 10 for female patients, 10 for male patients and three for either male or female patients.	61	53	6	6	5
Burgundy (Wales)	Covers a wide geographical area with a mix of urban and rural communities, serving a population of around 500,000. Mental health services are provided in three hospital sites and there are 75 beds in total. The main ward for data collection was mixed gender and had 21 beds, with one bed allocated for a child aged between 17 and 18 years (Child and Adolescent Mental Health Services).	43	48	6	6	0
Champagne (Wales)	Covers two contrasting areas: one urban and fairly ethnically diverse, the other rural and predominantly White British. Serves approximately 500,000 people through 2 psychiatric hospitals with 75 beds in total. The main ward used for intensive data collection at was mixed gender and had 19 beds.	41	48	4	6	0
Dauphine (England)	Covers an extremely densely populated and multicultural urban area. Serves approximately 750,000 people. Inpatient mental health services are provided from three hospital sites with 251 acute inpatient beds. The main ward for intensive data collection at this site was mixed gender and had 19 beds.	53	54	6	6	2
Languedoc (England)	Covers a largely rural area, serving a population of around 735,000 people. Provides inpatient adult services and there were 62 beds available across the two hospital sites. The main ward for intensive data collection at this site was a male ward with 22 beds.	50	47	3	6	1
Provence (England)	Covers a predominantly rural area, serving a population of around 1.5 million. Adult inpatient services are provided from 6 hospital sites and there were 290 acute inpatient beds. The main ward for intensive data collection was a mixed ward with 17 beds.	42	51	6	6	2
	Totals	290	301	31	36	10

We completed 31 research interviews with staff (target was 36), 36 with service users (target was 36); and nine with carers (target was 24).

Cross-site analyses will be presented for the four service user questionnaires (VOICE, RSA, STAR-P and ES) followed by a cross-site analysis of the two staff questionnaires (RSA and STAR-C).

### Service users

To explore cross-site differences one-way ANOVAs of all total score and subscales were conducted and revealed that there were no global differences across the sites for any of the four measures. Table 2 shows the mean item scores, alongside the parameters of significance for service user participants.

**Table 2:** Summary score for service user responses to the VOICE, RSA, STAR and ES scales

Scales and Subscales	One-way ANOVA Parameters	Artois^a^	Burgundy^a^	Champagne^a^	Dauphine^a^	Languedoc^a^	Provence^a^
Views on Inpatient Care (VOICE)							
Mean Total Score	F(5, 294) = 0.49, *p* = 0.787	49.43 (2.77)	45.69 (2.40)	51.56 (2.33)	48.77 (2.53)	49.04 (2.92)	48.55 (2.81)
Recovery Self-Assessment Scale (RSA)							
Life Goals	F(5, 284) = 0.14, *p* = 0.984	3.45 (0.15)	3.40 (0.15)	3.35 (0.14)	3.49 (0.14)	3.38 (0.17)	3.36 (0.16)
Involvement	F(5, 264) = 0.05, *p* = 0.999	3.08 (0.17)	3.07 (0.18)	3.11 (0.16)	3.11 (0.14)	3.16 (0.19)	3.06 (0.18)
Diversity of Treatment Options	F(5, 277) = 0.56, *p* = 0.734	3.16 (0.15)	3.31 (0.14)	3.01 (0.13)	3.29 (0.12)	3.12 (0.18)	3.15 (0.15)
Choice	F(5, 290) = 0.54, *p* = 0.748	3.06 (0.14)	3.40 (0.15)	3.25 (0.14)	3.23 (0.14)	3.19 (0.18)	3.26 (0.15)
Individually Tailored Services	F(5, 255) = 0.34, *p* = 0.891	3.17 (0.17)	3.28 (0.15)	3.22 (0.17)	3.19 (0.16)	3.12 (0.19)	2.99 (0.16)
Mean Total Score	F(5, 280) = 0.13, *p* = 0.989	3.21 (0.06)	3.32 (0.14)	3.24 (0.12)	3.30 (0.12)	3.23 (0.17)	3.20 (0.14)
Scale to Assess Therapeutic Relationships (STAR-P)							
Positive Collaboration	F(5, 288) = 0.45, *p* = 0.814	14.99 (0.94)	15.28 (0.93)	15.88 (0.82)	14.98 (0.97)	13.85 (1.13)	14.72 (1.02)
Positive Clinician Input	F(5, 290) = 0.99, *p* = 0.422	7.32 (0.47)	7.47 (0.46)	7.85 (0.46)	7.16 (0.44)	6.38 (0.56)	7.41 (0.48)
Non Supportive Clinician Input	F(5, 288) = 0.77, *p* = 0.569	6.77 (0.46)	7.19 (0.43)	7.62 (0.47)	7.04 (0.46)	7.71 (0.51)	7.75 (0.48)
Mean Total Score	F(5, 289) = 0.50, *p* = 0.778	29.02 (1.55)	30.00 (1.48)	31.35 (1.39)	29.17 (1.39)	27.93 (1.83)	29.58 (1.73)
The Empowerment Scale (ES)							
Self-esteem – self-efficacy	F(5, 287) = 1.16, *p* = 0.330	3.05 (0.10)	2.87 (0.13)	3.22 (0.10)	2.99 (0.10)	3.07 (0.10)	2.98 (0.11)
Power-powerlessness	F(5, 284) = 1.32, *p* = 0.257	2.47 (0.09)	2.40 (0.08)	2.54 (0.08)	2.32 (0.08)	2.52 (0.07)	2.56 (0.08)
Community activism and autonomy	F(5, 282) = 0.85, *p* = 0.515	3.22 (0.09)	3.34 (0.08)	3.29 (0.07)	3.25 (0.07)	3.41 (0.08)	3.25 (0.07)
Optimism and control over the future	F(5, 289) = 0.48, *p* = 0.788	2.89 (0.11)	2.95 (0.12)	2.98 (0.10)	2.98 (0.09)	3.12 (0.10)	3.00 (0.10)
Righteous anger	F(5, 287) = 0.59, *p* = 0.707	2.37 (0.11)	2.49 (0.11)	2.31 (0.11)	2.34 (0.08)	2.26 (0.11)	2.29 (0.10)
Total Score	F(5, 289) = 0.82, *p* = 0.539	2.85 (0.07)	2.81 (0.07)	2.93 (0.05)	2.80 (0.05)	2.92 (0.05)	2.85 (0.06)

^a^All values represent mean and standard error of the mean (SEM)

### Staff

For staff, one-way ANOVAs were conducted for the mean RSA and STAR-C total scores and the subscales (Table 3). There was a significant difference between the research sites in the mean RSA total score (F 5, 279) = 6.35, *p* <  0.001, η^2^ = 0.32) and the mean total score for the STAR-C (F 5, 273) = 3.02, *p* = 0.011, η^2^ = 0.23). There were also significant differences found in all of the mean item subscale scores of the RSA and the positive collaboration subscale for the STAR-C. Table 3 shows summary scores for staff.

**Table 3:** Summary scores for staff responses to the RSA and STAR-C

Scales and Subscales	One-way ANOVA Parameters	Artois^*^	Burgundy^*^	Champagne^*^	Dauphine^*^	Languedoc^*^	Provence^*^
Recovery Self-Assessment Scale (RSA)							
Life Goals	F(5, 273) = 4.44, *p* = 0.001 **	3.53 (0.08)	3.67 (0.11)	3.52 (0.08)	3.93 (0.07)	3.68 (0.10)	3.97 (0.09)
Involvement	F(5, 275) = 4.94, *p* < 0.001 **	3.15 (0.09)	3.05 (0.13)	2.85 (0.10)	3.47 (0.10)	3.25 (0.10)	3.46 (0.11)
Diversity of Treatment Options	F(5, 279) = 7.45, *p* < 0.001 **	3.21 (0.10)	3.42 (0.11)	3.06 (0.10)	3.72 (0.10)	3.39 (0.10)	3.81 (0.10)
Choice	F(5, 278) = 3.14, *p* = 0.009 **	3.47 (0.09)	3.72 (0.11)	3.46 (0.07)	3.73 (0.09)	3.84 (0.09)	3.79 (0.10)
Individually Tailored Services	F(5, 239) = 10.95, *p* < 0.001 **	3.29 (0.09)	3.34 (0.11)	2.92 (0.08)	3.81 (0.09)	3.32 (0.10)	3.75 (0.09)
Mean Total Score	F(5, 279) = 6.35, *p* < 0.001 **	3.36 (0.08)	3.45 (0.10)	3.21 (0.07)	3.74 (0.07)	3.52 (0.09)	3.76 (0.09)
Scale to Assess Therapeutic (STAR-C)							
Positive Collaboration	F(5, 274) = 2.42, *p* = 0.036 *	17.17 (0.35)	18.86 (0.39)	17.63 (0.42)	18.20 (0.33)	18.22 (0.36)	18.21 (0.48)
Positive Clinician Input	F(5, 272) = 1.53, *p* = 0.182	10.34 (0.18)	10.95 (0.18)	10.41 (0.21)	10.38 (0.15)	10.55 (0.20)	10.57 (0.19)
Emotional Difficulties	F(5, 270) = 1.91, *p* = 0.092	8.60 (0.20)	9.50 (0.21)	8.95 (0.27)	8.86 (0.23)	9.15 (0.21)	8.59 (0.38)
Mean Total Score	F(5, 273) = 3.02, *p* = 0.011 **	36.08 (0.55)	39.33 (0.66)	37.00 (0.76)	37.45 (0.52)	37.98 (0.62)	37.56 (0.77)

All values represent mean and standard error of the mean (SEM) * = Significant at *p* < 0.05 ** = Significant at *p* < 0.01

### RSA Total

**Fig. 2:**
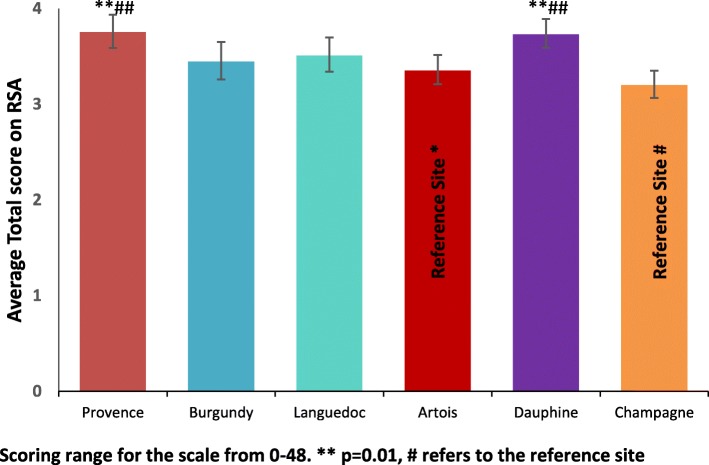
Mean Total RSA score for staff +_95% CI

When using Artois and Champagne as reference sites (the sites with the lowest scores) Provence and Dauphine sites scored significantly higher for the mean RSA total score indicating more recovery focused care (see Fig. 2). This scale measures some important perceptions that may have a significant effect on patient outcomes and concordance to care and collaboration with service users. Subsequent Tukey post-hoc tests revealed that staff in Artois (3.36, s.d. = 0.59) score significantly lower than Provence (3.76, s.d. = 0.56, *p* = 0.009, CI.95–0.73, − 0.07, Cohen’s d = 0.69) and Dauphine (3.74, s.d. = 0.53, *p* = 0.009, CI.95–0.70, − 0.06, Cohen’s d = 0.68). Staff in Champagne (3.21, s.d. = 0.46) score significantly lower than Provence (*p* <  0.001, CI.95–0.92, − 0.19, Cohen’s d = 1.07) and Dauphine (*p* <  0.001, CI.95–0.88, − 0.19, Cohen’s d = 1.07).

### STAR-C questionnaire

There were no significant differences in the staff responses across sites for the Positive Clinician Input subscale (F (5,272) = 1.53, *p* = 0.182, η^2^ = 0.16) and the Emotional Difficulties subscale (F (5,270) = 1.91, *p* = 0.092, η^2^ = 0.16) There were however significant differences found between sites for the Positive Collaboration subscale (F (5, 274) = 2.42, *p* = 0.036, η^2^ = 0.20) and the STAR-C Total score (F (5, 273) = 3.02, *p* = 0.011, η^2^ = 0.23).

### STAR-C positive collaboration

**Fig. 3:**
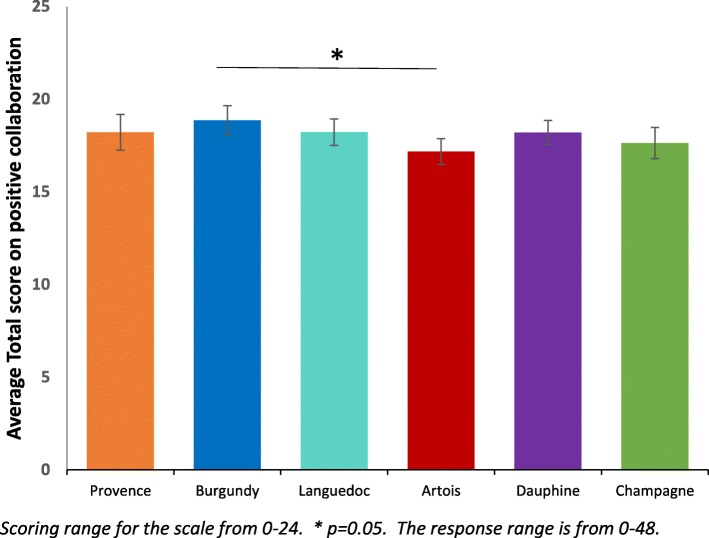
Mean Positive Collaboration subscale score for staff ±95% CI

Burgundy performs significantly better for the mean Positive collaboration subscale score than Artois (see Fig. 3). This scale measures some important perceptions around rapport and shared understanding of goals focused on mutual openness and trust. Subsequent Tukey post-hoc tests revealed that staff in Artois (17.17, s.d. = 2.65) score significantly lower on the subscale than Burgundy (18.86, s.d. = 2.57, *p* = 0.019, CI.95–3.20, − 0.18, Cohen’s d = 0.65). There were no significant differences between all of the other sites on this subscale.

### STAR-C Total

**Fig. 4:**
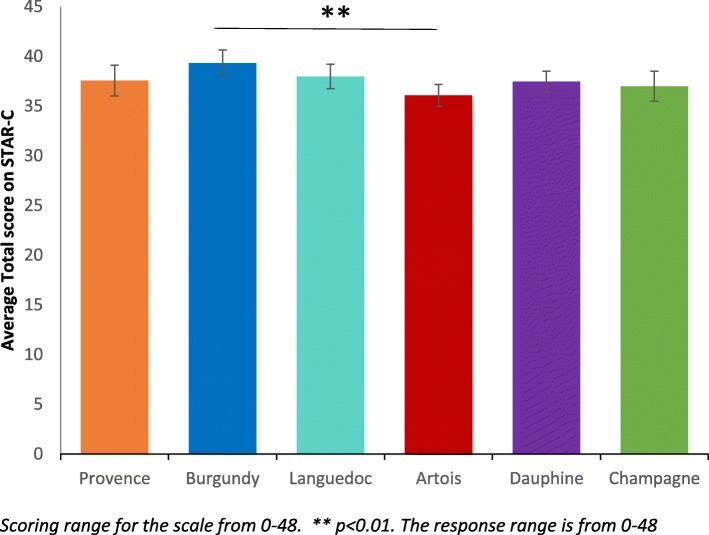
Mean STAR-C Total score for staff ±95% CI

Burgundy performs significantly better for the mean positive collaboration subscale score than Artois (see Fig. 4). This scale measures some important perceptions that may have a significant effect on patient outcomes and concordance to care and collaboration with service users. Subsequent Tukey post-hoc tests revealed that staff in Artois (36.08, s.d. = 4.18) score significantly lower on the subscale than Burgundy (39.33, s.d. = 4.31, *p* = 0.011, CI.95–5.76, − 0.75, Cohen’s d = 0.77). There were no significant differences between all of the other sites on total score.

### Correlations between outcome measures

Pearson’s correlations were used for the service user survey scores to determine if there were associations between responses on the four scales. Table 4 shows that there is a strong negative correlation between the RSA and VOICE (*r* = −.70, *n* = 285, *p* <  0.001). This shows that there is an inverse association between the recovery-oriented focus and the negative perception of quality of care amongst service users meaning when recovery-oriented focus was high the quality of care was viewed highly. There is also a positive correlation between the RSA and the STAR-P (*r* = .61, *n* = 282, *p* < 0.001), indicating an association between the recovery-oriented focus and ratings of the quality of therapeutic relationships amongst service users. There is also a strong negative correlation between the STAR-P and VOICE scale (*r* = −.64, *n* = 294, *p* <  0.001). There is also an inverse association between the quality of therapeutic relationships and the negative perception of quality of care meaning that when therapeutic relationships are scored highly the perception of quality of care is also scored highly. There are negligible relationships between the RSA and ES; STAR-P and ES and the VOICE and ES.

**Table 4:** Correlation analysis of the service user responses to the outcome scales (All sites)

Measures	N	r	Significance
RSA and VOICE	285	−0.690	< 0.001**
RSA and STAR-P	282	0.611	< 0.001 **
RSA and ES	282	0.085	0.153
STAR-P and ES	290	0.063	0.285
STAR-P and VOICE	294	−0.641	< 0.001**
VOICE and ES	295	0.055	0.349

**Correlation is significant at the 0.01 level

A Pearson’s correlation was completed at the global level with all participants to determine if there were associations for staff scores between the responses on the two questionnaire scales. This correlation were completed using pairwise deletion. There is a small to moderate correlation between the RSA and STAR-C (*r* = −.28, *n* = 279 *p* < 0.001).

When comparing the correlation between the RSA and STAR-C (see Table 5) there is a considerable amount of variability across sites. There is a large correlation in Burgundy (*r* = 0.50, *n* = 43, *p* = 0.001). There are moderate or small to moderate correlations in Artois (*r* = 0.28, *n* = 56, *p* = 0.034), Languedoc (*r* = 0.35, *n* = 47, *p* = 0.015) and Provence (*r* = 0.28, *n* = 56, *p* = 0.034). Only small correlations were found in Champagne (*r* = 0.16, *n* = 41, *p* = 0.331) and Dauphine (*r* = 0.35, *n* = 50, *p* = 0.015).

**Table 5:** Correlation analysis of the staff responses to the outcome scales (by site)

Measures	Parameter	Artois	Burgundy	Champagne	Dauphine	Languedoc	Provence
RSA and STAR-C	r	0.284	0.503	0.156	0.108	0.351	0.284
	Sig.	0.034^a^	0.001^b^	0.331	0.457	0.015^a^	0.034^a^
	N	56	43	41	50	47	56

^a^Correlation is significant at the 0.05 level ^b^ Correlation is significant at the 0.01 level

Across all of the six research sites staff score significantly higher than service users on the scale to assess therapeutic relationships. In Burgundy and Dauphine the same pattern is present across all of the subscales. Positive clinician input was scored higher by staff than service users across the six sites (see Table 2).

### Qualitative findings

Table 1 summarises the characteristics of each of the six meso-level case study sites and the types and quantity of data generated in each. Illustrative quotations used below are labelled with the initial of the site pseudonym; then ST, SU, or CA for staff, service user or carer; and their unique number, e.g. B-ST-001 (Burgundy-Staff-001).

#### Care planning and coordination

Staff across sites talked of the importance of collaborative care planning. Many also spoke of the value of plans being kept up-to-date with service users actively involved, and of plans being used as a way of collecting multidisciplinary contributions and of helping manage transitions between hospital and community. For example,

*“[B]ringing a person’s care all together really, so it’s like a standard to work around, that it’s all centred around the patient’s care, so everything works for them in the best way, I think.”* (L-ST-103)

However, staff, service user and carer interviews all revealed gaps between shared aspirations and realities, even where service users drew attention to receiving good quality care. Staff accounts of routine collaboration with service users in care planning contrasted with service user accounts which pointed to lack of involvement. In all sites some service users report that they were not involved in the planning of their care, were unaware of the content of their care plans or had not received copies, or did not feel a sense of care plan ownership. For example,

*“There isn’t a treatment plan. There’s no treatment, there’s just containment. Walking to the shop to get a newspaper isn’t treatment. There’s no therapy here.”* (C-SU-103)

Staff sometimes spoke of service users’ unwillingness or inability to collaborate in care planning, or of the barriers to collaborating brought about by the introduction of electronic records. Lack of a shared language was cited as a barrier in one inner city site (Dauphine). Staff in Burgundy said how the all-Wales CTP template was not well-suited to the short-term nature of acute hospital care with some domains (e.g., housing) emerging as higher priority than others.

*“I think I struggle with the principles [of CTP] and how that fits perhaps into the ward – the confusion that still exists is very much present in terms of the fundamentals of it.”* (B-ST-102)

Coherence and continuity in care across hospital and community interfaces were identified as important by many of those taking part, and examples of detailed and collaborative discharge planning involving staff and service users were given. Innovations were also described, such as ‘interim discharge summaries’. However, rapidly arranged discharges caused some concern with little time then available for considered planning, one service user recalled being ‘pulled in out of the blue’ to be told ‘right, you can go’ (P-SU-102).

Two types of care plan review were described: formal, typically weekly, multidisciplinary meetings chaired invariably by consultant psychiatrists and daily handovers where care on a more immediate basis was reviewed by staff. Formal ward rounds were described as key events by staff and as places where progress and plans could be reviewed in a multidisciplinary context. Service user views and experiences of these differed, within and across sites. For some they were helpful, serving as opportunities for catching up with psychiatrists and the whole multidisciplinary team.

*“Sometimes you’ve got a load of people in there and you sort of feel a bit like you’re on stage, you know like the spotlight’s on you, sort of thing. But yeah. I’ve had problems with ward rounds but more recently things have been OK, I’ve been able to sort of express myself more.”* (P-SU-104)

Some service users also described the opportunity to plan and prepare for formal ward round participation. Others spoke of limited time to fully consider their needs, of excessive jargon being used and of inflexibility over ward round scheduling.

#### Safety and risk

Assessing and managing risk were customarily seen by staff as central parts of the work of planning and providing care so that risk assessments were described as proliferating so much they were “*coming out of your eyeballs”* (P-ST-101). Formal ward round-based review meetings were named as a place for risks to be discussed although not necessarily in the presence of service users. Some staff also talked of the particular issues surrounding risk and decision-making in the care of service users who were detained. Risks mentioned by staff included those to self and others, with some also noting the dangers of over-estimating risks and the importance of attending to strengths and of positive risk-taking.

“*if you let the risk rule over the actual care plan then you’re never going to get anywhere*.” (L-ST-102)

Most service users talked of their safety being considered and attended to, sometimes giving specific examples of this in action (e.g., through removal of objects and the use of observations), even though risk assessments and management plans were often not actively discussed with them. Others did, however, talk of feeling unsafe in hospital and of asking for more staff.

#### Recovery

Definitions and understandings of recovery varied amongst staff, service users and carers, as did views of the role of hospitals in promoting this. Participants, in many cases, were also aware of the disparate meanings of ‘recovery’. Some staff (e.g., in Artois) viewed recovery as problematic in the inpatient context, saying that this raised expectations or was too poorly understood to help effective care planning.

“*I think it’s about being realistic as well. … certainly it’s about fostering hope, looking for things as well, and working towards those things, but in an acute ward where people can’t … leave [the ward]*” (A-ST-106)

In Languedoc, antipathy to the idea of recovery was reported by some staff who challenged both its meaning and utility. Most service users said that hospital had helped (e.g., to stabilise medication), though some complained of having been largely left to their own devices or subjected to containment. The use of tools to aid recovery (e.g., Recovery Star) were occasionally mentioned (e.g., in Burgundy), but in most cases these were either not deployed or were described as being more suitable beyond the acute hospital care context. Service users and carers revealed a range of views around recovery, from the cure of symptoms, to the prospects of life without medication, to the idea of coming to terms with difficulties.

*“getting rid of the voices and what I see. That’s my recovery”* (P-SU-101).

#### Personalisation

The term ‘personalisation’ was not a familiar one, with few revealing knowledge of personal budgets, “*It doesn’t mean anything, it just sounds like a made-up word*.” (L-SU-102).

In all settings there was recognition of the idea that care and services should be oriented to the individual. Whilst some staff talked of inpatient care as being person-centred there was also widespread recognition of the challenges to this (e.g., tensions between different approaches to providing care, the fact that staff only get to know people as patients, and the relative (un)availability of resources). Within and across sites there were differences in service user views and experiences of individually tailored care. Some were clear that hospital had been pivotal in their care, “*without this place it would be the end of me*” (C-SU-105). Others were equally clear that their care had not been personalised, or talked of their care at home being more personalised. Carers gave positive accounts of care provided although most remained uncertain about the term personalisation,

“*I guess personalisation means the way her treatment was personalised for her and I guess it was, because everyone is different and everyone needs different help, but I don’t really know what you mean.”* (P-CA-101)

## Discussion

The aim of this study was to identify factors that facilitate or hinder recovery-focused personalised care planning and coordination in acute inpatient mental health settings. The intention was to generate new theoretical knowledge and greater understanding of the complex relationships between collaborative care planning, recovery and personalisation.

Comparison and consideration of our survey results and interview data across sites provides some reason for optimism concerning the overall quality of mental health inpatient care but also indicators of areas where greater attention may be required.

We found no global differences across the six sites on the service user measures. The VOICE measure [41] examined service users’ perceptions of inpatient care and found marginally lower scores than the reference value [42]. However, the mean scores in all six research sites in this study were lower (so more positive) than those reported in a recently published study which examined different inpatient service models over a period from 2008 to 2010 [48]. We found that service users leaned towards a positive perception of the wards but there was wide variation within sites, suggesting a mix of views. These results converge with our research interview data showing service users being largely positive about their care, acknowledging being treated with dignity, respect and compassion. This was irrespective of legal status. Those carers interviewed also spoke positively about care provided and attitudes of staff.

Staff spoke of the challenges of collaborating on care planning with service users in severe mental distress or lacking insight and this is likely to include those formally detained. However, despite specific questions related to the legal status of services users, this was not explicitly identified as an issue perhaps reflecting the now high proportion of inpatients legally detained.

On ratings of the quality of therapeutic relationships, across all six sites staff consistently rated these relationships significantly more positively than did service users. The STAR-P measure used was initially designed for rating the one-to-one relationships that service users have with care coordinators in community teams [15] so it may be that, despite having a ‘named nurse’, the more dispersed nature of relationships with a number of ward staff over days and weeks, across shifts and 24-h care weaken any rating. Inpatient care also includes the greater likelihood of restrictions, limitations, rules and regulations necessary to provide a safe environment [49]. First- or second-hand experience of coercion and containment are also likely to be more prevalent in an inpatient setting [50]. Nevertheless, the need for further investigation to identify how positive relationships can be mutually achieved is indicated.

There was a strong perception across sites that staff were aware of policy drives to provide a greater focus on recovery, to provide respectful, compassionate and dignified care. Most staff articulated clear values and understandings reflecting core components of the focus on recovery as well as other initiatives that have been promoted in an attempt to improve inpatient services, such as the Royal College of Psychiatrists’ Accreditation for Inpatient Mental Health Services (AIMS) [51], Bright charity’s Star Wards [52] and most recently, the mental health nurse-led evidence-based intervention, SafeWards [53].

Staff participants suggested that severity of illness and/or lack of insight sometimes means that collaborative care planning is difficult to achieve, that there was often insufficient time to devote to this task, or that some service users were unwilling or unable to collaborate on care planning. Staff found it difficult to discuss care with service users especially where there was a mismatch in goals and expectations and limited advice on what a good care plan looks like or on how to identify achievable goals. Some of these barriers such as staff views on severity of illness have been found in other studies and highlighted in systematic reviews of barriers to involvement [54] and the consistency of this finding across our study sites can be read in a number of ways. First, it is undoubtedly the case that some people admitted to inpatient services are in severe distress and the process of discussing and negotiating a care plan in those first few days is unlikely to be a priority for them.

A second reading is that mental health professionals despite their claimed interest and support of involvement actually struggle to put this idea into practice and may need some guidance to achieve the aspiration of true collaboration. A possible contributor here was highlighted by both service users and staff and this relates to inflexible documentation and information technology on inpatient wards. In tandem these two elements prevent service users and staff writing care plans together as staff have to leave to type up a care plan once discussed, service users feel removed from the process and unable to alter the document which can often be presented to them without adequate explanation.

Services are also pressured to meet organisational demands and staff may simply not prioritise collaboration with service users. Service users report that time with staff is highly valued but for the most part was a limited resource. Time is an important and taken for granted feature of social life; it is used by individuals to impose order, understand and handle discontinuities [55, 56]. A universal expectation reported by staff and service users in this current study was that individual one to one time would provide the means for problem resolution, help establish rapport and trust and ultimately engender a sense of collaboration towards preferred goals. However, time was a scarce resource and organisational schedules were reported to quickly over-ride those of the service user and their primary nurse.

Interprofessional ward rounds were of critical importance to service users and staff alike as the site for discussion, planning and review. Service users and staff may experience the timetabling of ward rounds differently, for example there may be diverse perceptions of scheduling delays or contradictory understandings of what happened [57]. For service users in our study ward rounds involved anxious hours waiting to be called, followed by sometimes short but overwhelming or intimidating experiences in the meeting itself [58]. It was noted that few service users were adequately prepared on what to expect. Some told us they had expected to meet only the doctor but found themselves shown into a room full of unfamiliar faces, others felt that their contributions were not valued or that they had been poorly treated. For people who are already distressed and anxious about their treatment or future outcomes it seems ward rounds handled poorly can worsen their sense of efficacy and discourage attempts to achieve involvement.

Both staff and service users said that reviewing care plans in ward rounds would help mark progress towards agreed goals. This finding from our research interviews aligns with our quantitative survey showing that participants rated highly the recovery language used by staff and the regular monitoring of progress towards recovery goals. Additionally, the information needs of service users could be better met by helping them prepare for ward rounds, including determining expectations and the agenda. In addition it was suggested to us that service users be given summaries of ward round outcomes.

### Recovery, therapeutic relationships and care planning

The focus of recovery for many service users was around medication and symptom suppression (perhaps reflecting the primary focus of inpatient care) indicating a more ‘clinical’ as opposed to a ‘personal’ concept of recovery [9]. In some sites, there was greater ambivalence around the suitability or relevance of ‘recovery’ in inpatient care, particularly where people are very unwell. There may be tensions with working in recovery-focused ways when people are formally detained. It is possible however that this is the very time where a recovery-focused approach would be most powerful.

Our data on recovery shows convergence between results from standardised measures and findings from qualitative research interviews. Across five of the six sites service user participants rated highly the use of recovery language from workers and services alongside their perspective that workers believe that people can recover and participate in their own life choices. Service users also rated highly that there is regular monitoring of progress towards their recovery goals. Workers rated these items highly too suggesting that notions of recovery and therapeutic optimism were supported. Qualitative data indicate staff recognised the complex and individual nature of recovery. For example some staff saw a more recent orientation towards recovery focused care as representing the shift from previous authoritarian and prescriptive asylum based care to more collaborative models that encourage patient and family involvement.

There was a strong association amongst service users between their perceptions of recovery-oriented care and their perception of the quality of care on the ward. Likewise there were close correlations between the therapeutic relationships and the perception of quality of care. These findings were robust and consistent across all research sites. Whilst it is not possible to determine which factor might be influencing which, it does suggest an important interrelationship between service users’ subjective valuing of their relationships with staff, the quality of inpatient care and the recovery-focus of the service.

Across all sites staff consistently scored practices as more recovery-oriented than did service users. Our interviews, however, revealed ambivalence and a range of staff perspectives on recovery in line with previous research [59]. The concern that recovery creates ‘unrealistic’ expectations can perhaps be read as anxiety about what services have to offer to achieve this desired outcome. It may be that participants are simply acknowledging that recovery opportunities are hindered in settings where insufficient space is afforded to wider structural and social issues that give rise to and maintain mental distress. All participants appear to recognise the non-linear complex nature of recovery but place the emphasis differently.

One site that scored recovery highly, Dauphine, had made local attempts to introduce innovations such as service user-focused ‘This is Me’ care plans and short summary ‘management plans’, but these are in addition to standard documents and care plans, adding to workload. Interestingly, in Wales service user participants recognized that their goals were being monitored on a regular basis. This was appreciated to a lesser extent in England with just one site scoring this highly which may be a positive indication of the use of the structured care and treatment plan (CTP) approach in Wales.

### Safety and risk

Risk and safety remain key concerns for mental health workers [60] and issues around safety and risk are reported to be central to inpatient work for staff. In the mental health system more widely risk is constructed as an unwanted outcome arising from the actions or behaviours of individuals with mental health problems. In this sense risk is seen to emanate from the person who is seen as the chief agent of unwanted harmful behaviours. Harm does occur of course and mental health services appear to be chiefly concerned with harms from the person to themselves or others. For example, there are approximately 5500 suicides each year in the UK, 30% of which are known to mental health services [61–63]. Risk of suicide in the transition from inpatient care is now firmly established [64] and there is some suggestion that this risk has been transferred from inpatient to crisis resolution and home treatment services [65, 66]. Harm to others is a much rarer event but nevertheless is likely to have significant negative consequences for the victim, the individual with mental health problems and their family, and the wider system including individual workers such that risk averse practice is common [67]. The pressure to ensure safety and avoid blame appears to be omnipresent in mental health services.

Coherence and continuity in care across hospital and community interfaces is known to be important in delivering safe, supportive mental health care [68] and were identified as important by many of those taking part in this study, with examples of detailed and collaborative discharge planning involving staff and service users given. Innovations were also described, such as ‘interim discharge summaries’. However, participants also reported rapidly arranged discharges with little time for discussion or planning. Decisions on movement through phases of inpatient treatment will in part depend on the presenting symptomatology of the person, an assessment of their risk status, their needs for treatment and an assessment of their post discharge needs such as accommodation [69].

Staff acknowledged tensions around sensitive discussions and especially with people detained. Workers openly acknowledged that this was to avoid difficult conversations but others seemed less aware that in denying service users access to knowledge about their risk that they are effectively excluding people from participation in decisions about their care [70]. Previously we have noted that workers position risk assessment as legitimate work despite limitations in the predictive power of these judgements as one way of gaining normative certainty [71].

Here, unlike in the community study [18, 71], service users seemed to be more aware of their safety being considered and managed in that they understood why certain items were removed or restrictions were imposed. Some service users spoke of not feeling safe on wards as reported in previous studies [72, 73] and this needs to be considered in ongoing discussions and policy developments on safe staffing [74]. It remained a curious finding that while workers saw risk assessment as central to their efforts that they appear to largely exclude the service user from meaningful discussions about these.

### Personalisation

Drawing on the evidence presented here, personalisation is not widely recognized as a concept and not actively used in inpatient services by staff or service users, although there was wide discussion amongst staff of aiming to provide personal care or a personalised approach to care.

Staff spoke about some of the constraints and challenges in trying to work in a personalised way and these included a lack of resources, short ward stays, service users being formally detained, disagreements, risk behaviours, limited capacity, and a primary focus on medical treatment. It was recognised that to enable personalised care, it was necessary to have the time to get to know people as individuals and to provide some element of continuous care. Too often this was difficult to achieve in inpatient settings. Staff in the Welsh sites thought that the format of the CTP process and care plan was supportive of working in a personalised way and helped service users and staff get to know each other better.

Some service users were clear that their care was very personalized and that staff had considered their unique needs with several good examples provided. Others felt that inpatient care was more routine and standard for all and that individually tailored care was less possible in hospital, especially when people are detained. However, it was notable that some wards and staff were able to provide care in a more personalised way and support should continue to be given to achieve this everywhere. Personalisation is an integral component of a recovery-focused approach to mental health care and needs to be promoted and supported as such [75].

## Strengths and limitations

Achieving our target numbers for each grouping on the survey was challenging. Service user numbers were achieved but fell just short for staff. To achieve our target recruitment figures we approached all eligible participants meaning that our sample was not randomly selected. Despite considerable efforts we were unable to recruit sufficient numbers of carers. Researchers in the field reported how few carers visit wards, often preferring to meet service users elsewhere. The difficulties of involving carers in studies of inpatient mental health services has been reported elsewhere [76] and poses a particular challenge for researchers keen to include the views of family members and friends.

Due to the nature of the survey it is not possible to make comparisons between responders and non-responders as we had no access to data for non-participants. There was a moderate level of missing data for the RSA scale completed by service users, possibly due to some of the difficult language used and the community focus of the measure. As a consequence, more detailed analysis of covariations within the data was restricted by lack of power.

The interview data is rich and the framework method provided a time-consuming but structured and visible method of organising, analysing and comparing that data within and across sites. We believe the framework method and detailed presentation of results supports the transferability of these findings to other similar services. The involvement of service users and carers throughout the study as researchers and advisors has also provided added value to the study through additional viewpoints and interpretations.

## Conclusions

The findings of this cross-national, multi-site mixed methods study suggests positive practice is taking place within acute inpatient wards with evidence of a widespread commitment amongst staff to provide safe, respectful, compassionate care with strong values underpinning practice. Whilst ideas of recovery were evident amongst staff there was some uncertainty and discrepancy about the relevance of recovery ideals to inpatient care or the ability of people experiencing high levels of distress to engage in recovery-focused approaches. However, service users saw inpatient admissions as a necessary stage in stabilising their mental state, with medication an important component, and often appreciated the efforts that were made to keep them safe and to help them take the next tentative steps on their recovery. They also rate highly staff using recovery-focused language and values. Many spoke of care being personalised with examples given of staff being very responsive and considerate to particular needs or concerns. Carers often similarly described positive views of patient care. However, whilst service users valued the relationships they have with staff on the wards, they do not rate these as highly as staff. As discussed earlier, this is perhaps not surprising given all the tensions and anxieties associated with an inpatient stay, but this perhaps can best be summarised as ‘doing well, but could do better’.

Staff were clearly able to articulate the care planning processes and documentation required of them and described some of their frustrations with lengthy, unwieldy forms and at times distancing computerised systems that required more time in front of monitors than in conversation with service users. Most staff also spoke of their understanding and efforts to involve service users, and carers and families where possible, in the care planning process. However, most service users did not really appreciate the written care plan as an integral or important part of their experience and many did not have copies or could not find them. The majority of service users did not feel they had been genuinely involved in the process. Unfortunately, in relation to service users receiving sufficient time with nursing staff and being involved in planning their care, very little progress appears to have made since the report of the Healthcare Commission of nearly a decade ago [25].

Issues of risk and safety are ever-present in mental health services and it was clear that this was central to the work of staff, whilst they displayed an awareness of the sensitivities and challenges involved. Service users, and carers, were often aware of efforts being made by staff to keep them safe. However, involvement of service users in discussions about personal risk factors and safety is challenging and requires greater training and support to encourage staff to develop the skills and confidence to undertake such sensitive and important work with confidence.

## Additional file

Additional file 1: Reliability Assessment.docx showing additional reliability checks for the internal consistency of the scales and subscales. (DOCX 23 kb)

## Abbreviations

ANCOVA: Analysis of Covariance
ANOVA: Analysis of Variance
CPA: Care Programme Approach
CQC: Care Quality Commission
CTP: Care and Treatment Planning
ES: Empowerment Scale
LEAG: Lived Experience Advisory Group
MANCOVA: Multivariate Analysis
MDT: Multidisciplinary Team
MRC: Medical Research Council
NHS: National Health Service
NRES: National Research Ethics Service
RSA: Recovery Self-Assessment
SSC: Scientific Steering Committee
STAR-C: Scale to Assess the Therapeutic Relationship – Clinician version
STAR-P: Scale to Assess the Therapeutic Relationship – Patient version
SUGAR: Service User and Carer Group Advising on Research
SUPA: Service User Project Assistants
SURA: Service User Researcher Assistants
UK: United Kingdom
VOICE: Views of Inpatient Care Scale
