# Supplementary material for: Recovery-focused mental health care planning and co-ordination in acute inpatient mental health settings: a cross national comparative mixed methods study
Source: BMC Psychiatry. 2019 Apr 16;19:115. doi: 10.1186/s12888-019-2094-7 (PMC6469117; doi:10.1186/s12888-019-2094-7)
Supplement: Supplementary file 1 — Reliability Assessment.docx showing additional reliability checks for the internal consistency of the scales and subscales. (DOCX 23 kb) [file 12888_2019_2094_MOESM1_ESM.docx]

**Supplementary Data**

The internal consistency of all scales and subscales were initially assessed using Cronbach’s alpha based on complete cases for each scale or subscale. Internal consistency was evaluated separately for Service Users and Staff. An alpha level of 0.7 or above is often considered acceptable for the purpose of scale construction (Kline, 2000). The Cronbach’s alphas in Supplementary Table 1 suggest that some subscales were not performing acceptably (see asterisks). However, Cronbach’s alpha is strongly affected by the number of items in the scale and may underestimate internal consistency when the number of items is small. Therefore, alternative estimates of internal consistency were examined using mean item-total correlations and the Spearman-Brown prediction value. Mean item-total correlations are often used to examine internal consistency for very short scales. A value of 0.2 or 0.3 or above is considered acceptable (Field, 2005). We used the Spearman-Brown prediction formula to estimates values of Cronbach’s alpha if the number of items in the subscale were increased to 10 (while retaining the same strength of relationships between items). This provides estimates in internal consistency that are more directly comparable to each other. The mean item-total correlations suggest that only two empowerment subscales (Power-Powerlessness; Righteous Anger) failed to meet the higher threshold of acceptability (0.3) but both met the lower threshold (0.2), while the Spearman-Brown prediction values suggest all short scales and subscales (< 10 items) would demonstrate acceptability for internal consistency if they were extended to 10 items. Based on this analysis we conclude that all scales demonstrated adequate internal consistency for current purposes, although two of the empowerment subscales merit further psychometric development in future research.

**Supplementary Table 1:** Additional reliability checks for the internal consistency of the scales and subscales

| **Participant** | **Scale or Subscale** | **Number of cases** | **Number of items in Scale** | **Cronbach's α** | **Mean item total correlation** | **Spearman-Brown prediction value** |
| --- | --- | --- | --- | --- | --- | --- |
|  | **Views on Inpatient Care (VOICE)** |  |  |  |  |  |
| Service user | VOICE Total | 215 | 19 | 0.924 | 0.607 | - |
|  | **Recovery Self-Assessment Scale (RSA)** |  |  |  |  |  |
| Service user | RSA Total | 103 | 36 | 0.976 | 0.721 | - |
|  | Life Goals | 179 | 11 | 0.931 | 0.714 |  |
|  | Involvement | 163 | 8 | 0.913 | 0.717 | 0.954 |
|  | Diversity of Treatment Options | 172 | 6 | 0.814 | 0.576 | 0.946 |
|  | Choice | 217 | 6 | 0.808 | 0.573 | 0.944 |
|  | Individually Tailored Treatment options | 159 | 5 | 0.849 | 0.659 | 0.966 |
| Staff | RSA Total | 186 | 36 | 0.945 | 0.559 |  |
|  | Life Goals | 246 | 11 | 0.861 | 0.553 |  |
|  | Involvement | 225 | 8 | 0.849 | 0.589 | 0.919 |
|  | Diversity of Treatment Options | 225 | 6 | 0.774 | 0.520 | 0.932 |
|  | Choice | 254 | 6 | 0.676^*^ | 0.418 | 0.893 |
|  | Individually Tailored Treatment options | 253 | 5 | 0.711 | 0.472 | 0.925 |
|  | **The Empowerment Scale (ES)** |  |  |  |  |  |
| Service user | Empowerment Total | 255 | 28 | 0.817 | 0.350 |  |
|  | Self-esteem- self efficacy | 272 | 9 | 0.911 | 0.695 | 0.911 |
|  | Power- powerlessness | 271 | 7 | 0.556* | 0.281 | 0.790 |
|  | Community activism and autonomy | 276 | 5 | 0.579* | 0.343 | 0.873 |
|  | Optimism & control over the future | 275 | 4 | 0.704 | 0.493 | 0.935 |
|  | Righteous anger | 281 | 3 | 0.403* | 0.250 | 0.825 |
|  | **Scale to Assess Therapeutic Relationships (STAR)** |  |  |  |  |  |
| Service user | STAR-P Total | 264 | 12 | 0.890 | 0.604 |  |
|  | Positive Collaboration | 279 | 6 | 0.924 | 0.780 | 0.980 |
|  | Positive Clinician Input | 282 | 3 | 0.723 | 0.545 | 0.948 |
|  | Non-supportive Clinician Input | 284 | 3 | 0.668* | 0.643 | 0.934 |
| Staff | STAR-C Total | 263 | 12 | 0.811 | 0.480 |  |
|  | Positive Collaboration | 269 | 6 | 0.814 | 0.586 | 0.946 |
|  | Positive Clinician Input | 268 | 3 | 0.560* | 0.461 | 0.899 |
|  | Emotional Difficulties | 273 | 3 | 0.633* | 0.374 | 0.924 |

Key: STAR-P: Scale to Assess Therapeutic Relationship – Patient version; STAR-C: Scale to Assess Therapeutic Relationship – Clinician version

**References**

Field, A., (2005). Discovering Statistics Using SPSS (2nd ed). London: Sage

Kline P. (2000). Handbook of psychological testing (2nd ed). London: Routledge.
